# Single nucleotide polymorphisms are typical for tick-borne encephalitis and West Nile viruses during triple natural mixed infections in Blyth’s reed warbler (Acrocephalus dumetorum)

**DOI:** 10.18699/vjgb-26-12

**Published:** 2026-03

**Authors:** E.P. Ponomareva, V.A. Ternovoi, E.V. Protopopova, N.L. Tupota, V.B. Loktev

**Affiliations:** State Research Center of Virology and Biotechnology “Vector” , Koltsovo, Novosibirsk Region, Russia; State Research Center of Virology and Biotechnology “Vector” , Koltsovo, Novosibirsk Region, Russia; State Research Center of Virology and Biotechnology “Vector” , Koltsovo, Novosibirsk Region, Russia; State Research Center of Virology and Biotechnology “Vector” , Koltsovo, Novosibirsk Region, Russia; State Research Center of Virology and Biotechnology “Vector” , Koltsovo, Novosibirsk Region, Russia Institute of Cytology and Genetics of the Siberian Branch of the Russian Academy of Sciences, Novosibirsk, Russia

**Keywords:** tick-borne encephalitis virus, West Nile virus, single nucleotide polymorphism, viral genome, high-throughput sequencing, garden warbler, orthoflaviviruses, вирус клещевого энцефалита, вирус Западного Нила, однонуклеотидный полиморфизм, вирусный геном, высокопроизводительное секвенирование, садовая камышовка, ортофлавивирусы

## Abstract

Wild bird species contribute significantly to the rapid geographic dissemination of tick-borne encephalitis viruses (TBEV) and West Nile virus (WNV), facilitating the establishment of new natural foci of these orthoflaviviruses. However, the impact of TBEV and WNV population variability on shaping these foci, as well as the potential emergence of new human-pathogenic viral variants, remain underexplored. This study aimed to assess the genetic heterogeneity of TBEV (Siberian and Far Eastern genotypes) and WNV, isolated simultaneously from the tissues of a single garden reed warbler (Acrocephalus dumetorum) collected in the suburbs of Tomsk. The methods of viral strain isolation on various cell cultures were used in combination with a whole-genome analysis of isolates through traditional and high-throughput sequencing (NGS) methods. Consensus full-genome nucleotide sequences of the viruses were obtained by Sanger sequencing and compared with those obtained by NGS, with single nucleotide substitutions (single nucleotide variants, SNVs) accounting for 2 % or higher within the population under study. Our findings revealed single nucleotide polymorphisms (SNPs) associated with both synonymous and non-synonymous nucleotide substitutions, primarily located within the non-structural protein genes of TBEV and WNV. Notably, recombination events were not detected in the genomes of isolated orthoflaviviruses. The WNV isolate, Tomsk/bird/2006/A4, and the TBEV isolates, PT12 and PT122, obtained from A. dumetorum, exhibited heterogeneous viral populations, with SNVs ranging in frequency from 1.75 to 19.88 % for WNV and from 2.08 to 23.73 % for TBEV. Most identified SNPs shared similar nucleotide substitutions in the genomes of already known strains of TBEV and WNV, suggesting that these SNVs could play a crucial role in viral adaptation and underscore the genetic and phenotypic diversity of these viruses in nature.

## Abstract (ru)

Вовлечение различных видов диких птиц в формирование природных очагов вирусов клещевого энцефалита (ВКЭ) и Западного Нила (ВЗН) обеспечивает быстрое распространение этих ортофлавивирусов в различных географических районах и формирование новых природных очагов данных инфекций. Однако роль популяционной вариабельности ВКЭ и ВЗН в формировании природных очагов, возможность появления (селекции) новых вирусных вариантов, патогенных для человека, в этих природных очагах остаются еще недостаточно изученными. Цель настоящего исследования – оценить популяционную гетерогенность геномов ВКЭ сибирского и дальневосточного генотипов и ВЗН, которые были одновременно выделены из тканей одной особи садовой камышовки (Acrocephalus dumetorum), отловленной в природных ландшафтах пригорода Томска. С этой целью были использованы методы выделения вирусных штаммов на различных культурах клеток в сочетании с анализом полных вирусных геномов полученных изолятов, определенных методами традиционного и высокопроизводительного секвенирования (NGS). Консенсусные полногеномные нуклеотидные последовательности вирусов получали секвенированием по Сэнгеру и сравнивали с последовательностями, полученными методом NGS, с однонуклеотидными заменами (single nucleotide variant, SNV) 2 % и выше, в пределах изучаемой популяции. Обнаруженный однонуклеотидный полиморфизм (SNP) ассоциировался как с синонимичными, так и несинонимичными нуклеотидными заменами преимущественно локализующихся в генах неструктурных белков ВКЭ и ВЗН. При этом возможных рекомбинационных событий в геномах изолированных ортофлавивирусов обнаружить не удалось. Результаты показали, что изоляты ВЗН Tomsk/bird/2006/A4, ВКЭ PT12 и PT122, выделенные из A. dumetorum, представлены гетерогенными вирусными популяциями, в которых обнаруживаются множественные SNV, возникающие с частотой от 1.75 до 19.88 % для ВЗН и от 2.08 до 23.73 % для ВКЭ. Для большинства выявленных SNP найдены аналогичные нуклеотидные замены в геномах уже известных штаммов ВКЭ и ВЗН, что может свидетельствовать о важной роли этих SNV-спектров в вирусной адаптации и обеспечении генетического и фенотипического разнообразия этих вирусов в природе.

## Introduction

Tick-borne encephalitis virus (Orthoflavivirus encephalitidis) and West Nile virus (Orthoflavivirus nilense) belong to the Flaviviridae family, genus Orthoflavivirus (Current ICTV Taxonomy Release; Postler et al., 2023). Tick-borne encephalitis virus (TBEV) and West Nile virus (WNV) are capable of inducing severe human diseases, potentially resulting in significant damage to the central nervous system (Worku, 2023; Singh et al., 2024). These viruses establish natural foci, with human infection commonly resulting from mosquito (WNV) or tick (TBEV) bites. The natural foci of TBEV are characteristic of northern Eurasia, whereas WNV exhibits a near-global distribution (Pustijanac et al., 2023; Simonin, 2024). Co-circulation of these two orthoflaviviruses has been observed in southern Western Siberia (Ternovoi et al., 2004; Kononova et al., 2006).

TBEV and WNV are characterized by genetic diversity, with at least five main genotypes described for TBEV and at least nine genotypes for WNV (Dai et al., 2018; Kozlova et al., 2018; Simonin, 2024). The level of nucleotide differences between different genotypes can reach 18–20 %. Siberian and Far Eastern TBEV genotypes, as well as WNV genotype 1a, are prevalent within the Siberian region.

Modern high-throughput sequencing methods enable the detection of SNPs in relatively small viral populations. A study examining the presence of SNVs in populations of the EK-328 TBEV strain, which had been adapted to various cultivation conditions, and its cloned variants revealed that SNVs occur at a frequency of approximately 1 % in populations of TBEV laboratory strains (Litov et al., 2018). This finding allowed us to assume that minor SNVs of TBEV could facilitate microevolution and rapid adaptation of the viral population to environmental changes, even under laboratory conditions. This hypothesis was confirmed by the studies on the variability of the genome of the C11-13 TBEV strain of the Siberian genotype when cultivated in the laboratory (Ternovoi et al., 2024). The presence of single nucleotide polymorphism has also been described in the genomes of Zika, dengue, Japanese encephalitis viruses, and WNV (Kaiser et al., 2019; Zaráte et al., 2019; Borda et al., 2021). However, attempts to attenuate West Nile virus using SNPs specific to the Japanese encephalitis virus E protein gene yielded no positive results. A suggestion was made that each orthoflavivirus species possesses an unique SNP profile.A previous study (Mikryukova et al., 2014; Moskvitina et al., 2014; Korobitsyn et al., 2021) showed the involvement of 42 out of 60 bird species studied in the circulation of TBEV and WNV in the Tomsk and Novosibirsk regions. Additionally, a significant percentage of both birds (up to 39 %) and ticks removed from the birds (2.13 %) exhibited evidence of mixed infections. Given the diversity of bird species that circulate TBEV and WNV, we should consider how these orthoflaviviruses adapt to different hosts. The involvement of various wild bird species in the formation of natural foci of TBEV and WNV was assumed to facilitate the rapid spread of these orthoflaviviruses across different geographic areas, leading to the establishment of new natural foci of these infections. Further research is needed to elucidate the impact of TBEV and WNV population variability on the formation of natural foci and the potential emergence (selection) of new humanpathogenic viral variants within these natural foci.

This study aimed to assess the population heterogeneity of the WNV and TBEV genomes found in the tissues of one garden reed warbler captured in the suburbs of Tomsk. To achieve this, we employed methods for isolating viral strains from various cell cultures, along with an analysis of complete viral genomes from the obtained isolates. This analysis was conducted using both traditional and high-throughput sequencing techniques

## Materials and methods

Samples studied. A 10 % homogenate, combining spleen and liver tissue, was prepared from a garden reed warbler (Acrocephalus dumetorum) captured in the Tomsk region in 2006 (Mikryukova et al., 2014). Orthoflavivirus isolation was achieved using cells culture porcine embryo kidney (PEK) and the Aedes albopictus mosquito (C6/36) obtained from the cell culture collection of the State Research Center of Virology and Biotechnology “Vector”. The cell cultures were grown in DMEM/F12 medium (“Vector”, Russia) supplemented with 10 % fetal bovine serum (Gipco, USA) and 80 μg/ml gentamicin sulfate at 37 °C for PEK cells and 28 °C for C6/36 cells.

The infectious activity of the viral isolates was determined by the development of the cytopathogenic effect (CPE). For this purpose, 105 cells of PEK were seeded into 96-well culture microplates in a volume of 50 μl per well and infected with virus-containing material. The viruses were titrated in DMEM/F12 medium containing 2 % fetal bovine serum in a volume of 100 μl per well. Following a five-day period, the viral infectious titer was calculated as described previously (Svyatchenko et al., 2021).

Enzyme immunoassay. TBEV and WNV antigens were detected in the culture medium using mouse monoclonal antibodies 13F6 (against TBEV) and 9E2 (against WNV) as at antigen capture (Razumov et al., 2005; Shanshin et al., 2024). Bound antigens were detected using monoclonal antibodies 10H10 (TBEV) and 5H6 (WNV) labeled with biotin and streptavidin-peroxidase conjugate (ICN, USA), as described previously (Korobitsyn et al., 2021).

Sample preparation. Post-treatment with benzonase (Law et al., 2013), the total RNA was extracted using the Extract RNA reagent (Eurogen, Russia), according to the manufacturer’s protocol. The first DNA strand was constructed using the MMLV RT kit (Eurogen, Russia), according to the manufacturer’s instructions. PCR was performed using “BioMaster LR HS-PCR” (BioLabMix, Russia), with primers for the detection of TBEV and WNV RNA, respectively (Supplementary Materials 1 and 2)1. PCR mode (C1000 amplifier, Bio-Rad, USA) was as follows: 94 °C for 10 s, 58 °C for 20 s, and 72 °C for 30 s (40 cycles), followed by 72 °C for 7 minutes.

Supplementary Materials are available in the online version of the paper: https://vavilov.elpub.ru/jour/manager/files/Suppl_Pon_Engl_30_1.pdf


Electrophoretic analysis and isolation of viral DNA fragments from gel. Amplification products were analyzed in a 2 % agarose gel in TAE x1 buffer (40 mM Tris, 1 mM Na2EDTA). The diaGene kit (Dia-M, Russia) was used to isolate the amplification products from the agarose gel.Determination of nucleotide sequences of viral cDNA. Nucleotide sequences of amplification products were determined using an ABI 3130xl automated genetic analyzer (Applied Biosystems, USA) with the BigDye reagent kit. We also used the Terminator v 3.1 Cycle Sequencing Kit (Applied Biosystems, USA), according to the manufacturer’s instructions. The alignment of nucleotide sequences was performed using the Lasergene 7 (DNASTAR) application.

First-strand cDNA synthesis for NGS was performed using the NEBNext® Ultra Directional (NEB) module. Secondstrand synthesis was performed using the UMI Second Strand Synthesis Module for QuantSeq FWD (Illumina, Lexogen). Cutadapt (version 1.18) and SAMtools (version 0.1.18) were used to remove Illumina adapters and reformat the reads. The contigs were assembled de novo using the MIRA assembler (version 4.9.6).

The nucleotide sequences for comparison were taken from the GenBank database. Multiple nucleotide sequence alignments were performed using the AlignX application within the Vector NTI 11 software package (InforMax). The analysis of the obtained nucleotide sequences was performed using the Unipro UGENE v.1.30 and MEGA 7/10 programs (Kumar et al., 2018). The phylogenetic trees were calculated using the maximum likelihood method using 1,000 bootstrap replicates.

## Results

**Fig. 1.**
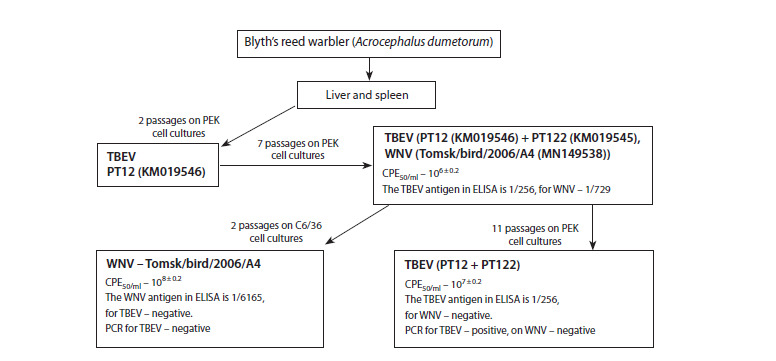
Scheme for isolating TBEV and WNV isolates from the combined homogenate of the spleen and liver of the garden warbler on cell cultures As cell culture passages were performed, each block marked in the diagram was subjected to determination of infectious activity, determination of the presence of viral antigen in ELISA using monoclonal antibodies, PCR, and sequencing of the sample using Sanger and high-throughput sequencing methods, as described in the “Materials and methods” section.

This study utilized a combined liver and spleen sample from the garden warbler (A. dumetorum) caught in the Tomsk region in 2006 (Mikryukova et al., 2014). Figure 1 illustrates the process of isolating viral isolates from this sample using C6/36 and PEK cell cultures

The first instance of a cytopathogenic effect, attributable to TBEV replication, appeared after just two passages. However, after seven additional passages, the antigen and genetic material of WNV and TBEV were detected in the sample. Passaging the infectious material in mosquito C6/36 cells allowed us to obtain a pure culture of WNV. Supplementary passages of identical PEK cell material resulted in the eradication of the WNV population and the establishment of a stable TBEV population.

The genomic sequencing of the samples allowed us to determine the nucleotide sequences for two TBEV isolates (Tomsk- PT12 and Tomsk-PT122) and one WNV isolate (Tomsk/ bird/2006/A4). The phylogenetic analysis of whole-genome sequences revealed that isolate Tomsk-PT12 belonged to the Far Eastern genotype of TBEV, while isolate Tomsk-PT122 represented the Siberian genotype of TBEV. The Tomsk/ bird/2006/A4 isolate was genotyped as a virus belonging to genotype Ia of WNV (Supplementary Material 3).

**Fig. 2.**
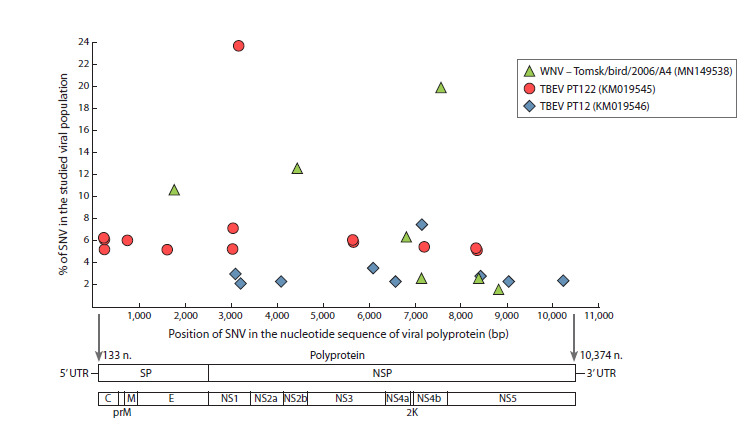
Mapping of single nucleotide substitutions in the TBEV and WNV genomes using high-throughput sequencing.

High-throughput sequencing analysis of nucleotide sequences identified SNVs within viral populations. Figure 2 presents the single nucleotide substitutions identified in the three viral populations, with a frequency threshold of 1.75 % or greater. Eastern genotype of TBEV was completely devoid of SNVs in the genomic region encoding the structural proteins. The most significant number of SNPs was detected in this region of the genome for the Siberian genotype of TBEV.

Our findings indicate the presence of heterogeneous viral populations in the WNV strains (Tomsk/bird/2006/A4) and the TBEV strains (Tomsk-PT12 and Tomsk-PT122) isolated from A. dumetorum. These populations contain multiple SNVs, with frequencies ranging from 1.75 to 19.88 % for WNV and from 2.08 to 23.73 % for TBEV (see the Table). The identified SNPs are mapped throughout the viral genome, both in the genes of structural viral proteins and in the genes of non-structural proteins. At the same time, isolate Tomsk-PT12 of the Far Eastern genotype of TBEV was completely devoid of SNVs in the genomic region encoding the structural proteins. The most significant number of SNPs was detected in this region of the genome for the Siberian genotype of TBEV

**Table 1.**
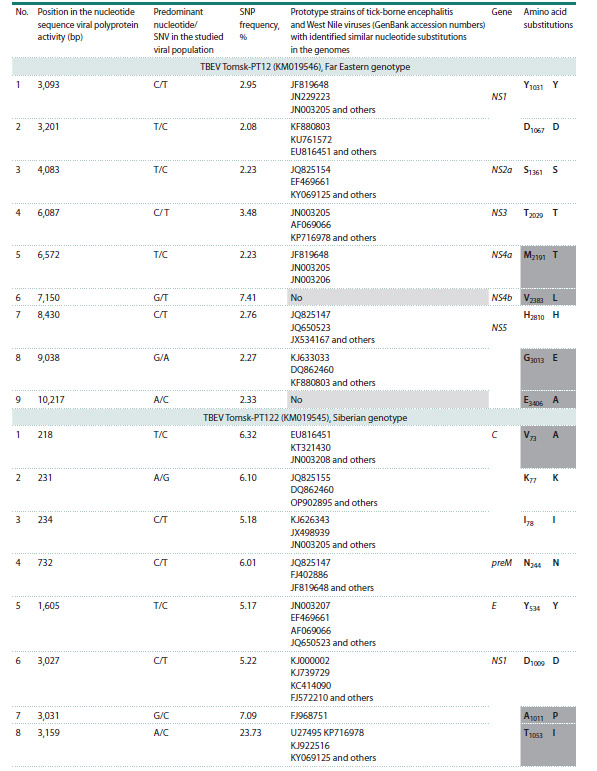
Single nucleotide substitutions in the genomic RNA of WNV and TBEV during associated infection in A. dumetorum Notе. Non-synonymous nucleotide substitutions are highlighted with a dark gray background; a light gray background indicates the absence of prototype strains with identified similar nucleotide substitutions in the genomes. The amino acid designations are given under the generally accepted international single-letter code.

**Table 1end.**
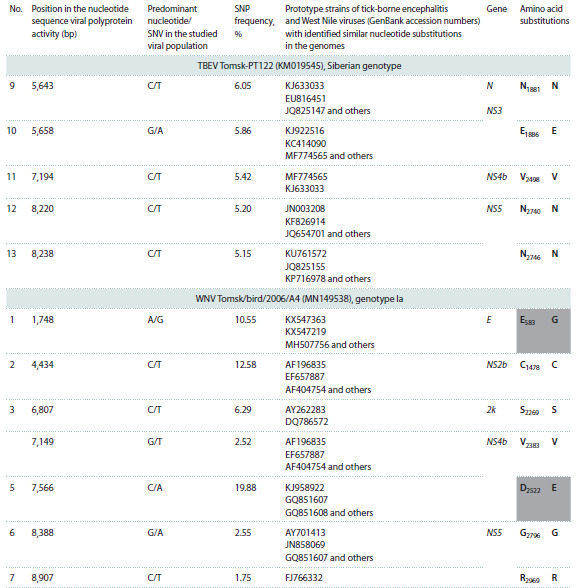
Table 1end.

Of further interest is the observation that all 29 SNPs identified in the three orthoflavivirus isolates proved to be original. These results indicate that the detected SNV spectrum is characteristic and specific for the studied TBEV and WNV viral populations within a single infected host. This finding is indirectly confirmed by previously obtained data on the mismatch of SNVs for Japanese encephalitis virus and WNV (Kaiser et al., 2019). Whole-genome sequencing of these viruses did not detect evidence of recombination across extensive fragments of the isolated TBEV and WNV strains found in a single wild bird specimen. The unique SNV pattern for all three isolates confirms the absence of recombination during the simultaneous circulation of three viruses in a single host.

The Siberian genotype TBEV isolate Tomsk-PT122 exhibited the highest number of detected SNPs (13), while the lowest number (7) was observed in WNV. More than 60 % of SNPs were mapped in genes encoding non-structural viral proteins. The mean SNP frequency, relative to the consensus genome, was 3.1 % for the Tomsk-PT12 isolate of TBEV (Far Eastern genotype), 7.1 % for the Tomsk-PT122 isolate of TBEV (Siberian genotype), and 8.0 % for the WNV genotype 1a isolate (see the Table).

Notably, the identified SNPs exhibited analogous confirmed substitutions within the genomes of the established TBEV and WNV strains. Only theSNP7150NS4b and SNP10217NS5 proteins mapped for the Far Eastern genotype of TBEV had no analogs among the known TBEV genomes. Hence, the presence of identical nucleotide substitutions across the genomes of previously described viral isolates indicates a non-random nature of SNV occurrence, potentially contributing to the genetic variability of these flaviviruses

## Discussion

It is known that isolates of various RNA-containing viruses typically represent a heterogeneous population of closely related variants, often referred to as quasispecies (Eigen et al., 1988; Holland et al., 1992; Domingo, Holland, 1997; Domingo et al., 2012; Karbowiak et al., 2016). Viral populations are characterized by a high number of viral particles, with sizes potentially reaching 1010–1012 virions or higher per infected macroorganism (Marí Saéz et al., 2015; Diallo et al., 2016; Thorson et al., 2016; Domingo et al., 2021). Moreover, a minimal quantity of viral particles is sufficient for infection of a susceptible organism. Further replication of RNA-containing viruses in the host organism ensures the formation of a heterogeneous viral population.

Predominantly, the extensive variability of viral RNA genomes arises from the high level of RNA polymerase errors and nucleotide substitutions, deficiencies in error correction mechanisms, and the action of selective forces exerted during replication within the host.

Previous findings reported the circulation of WNV and TBEV in the urban and suburban biotopes of Tomsk (Moskvitina et al., 2008; Chausov et al., 2009). Notably, the natural foci of these infections in this region involved over 42 diverse wild bird species (Mikryukova et al., 2014; Moskvitina et al., 2014; Korobitsyn et al., 2021). At the same time, the genetic markers of both TBEV and WNV were identified in 1.7 % of the examined samples. The literature also reports cases of mixed infections caused by different TBEV genotypes (Bezrukova et al., 2008; Kovalev et al., 2008; Kozlova et al., 2010; Pogodina et al., 2012; Bezrukov et al., 2015). Alternatively, the prevalence of diverse TBEV subtypes identified in ixodid ticks ranges from 4.4 to 15 %.

The level of genetic differences between the European, Siberian, and Far Eastern genotypes of TBEV can reach 18–20 % (Ternovoi et al., 2007). TBEV and WNV belonging to different types of orthoflaviviruses demonstrate significant genomic heterogeneity, with sequence divergence frequently exceeding 28–32 %. The distinct genetic profiles of these two orthoflavivirus types enabled the identification of at least five main genotypes for TBEV and nine for WNV (Dai et al., 2018; Kozlova et al., 2018; Simonin, 2024). The various genotypes of these viruses tend to be associated with distinct natural foci situated in different parts of the world.

Recently accumulated data suggest a possible joint circulation and widespread distribution of various orthoflaviviruses in new regions. This trend is corroborated by the circulation of TBEV and WNV within the urban and suburban biotopes of Tomsk. The discovery of the joint circulation of several orthofaviviruses in an individual garden reed warbler clearly demonstrates the distinctive epidemiological features of these viruses in southern West Siberia.

The identification of a whole set of SNVs in populations of the isolates (Tomsk/bird/2006/A4 WNV, Tomsk-PT12, and Tomsk-PT122 TBEV) in the tissues of one wild bird indicates a pronounced heterogeneity of these viral populations. The possibility of random nucleotide substitutions in genomic RNA cannot be excluded. However, most of the identified SNPs share similar nucleotide substitutions with those found in the genomes of already-known WNV and TBEV. The genetic diversity of these flaviviruses could be limited by the number of SNPs, which predetermines the scope of genetic variation of these orthoflaviviruses within natural foci. Mapping of the original SNV patterns characteristic of each of the three different isolates of orthoflaviviruses under study, which were circulating simultaneously in a single infected host, was performed. The results demonstrate that the viral population heterogeneity is maintained within the Siberian and Far Eastern genotypes of TBEV and the genotype Ia of WNV via independent mechanisms. It is worth noting that we failed to identify the signs of new recombination events during the assembly and analysis of the consensus genomes of these viruses (Supplementary Material 4).

Our findings reveal pronounced heterogeneity of the flavivirus genomic RNA population, a characteristic preserved during triple flavivirus coinfection of a single host. The presence of multiple SNVs within even a limited viral population of orthoflaviviruses is likely to serve as a significant mechanism for the formation of new TBEV and WNV genovariants, even during the replication of these viruses in the tissues of the infected host.

## Conclusion

The evaluation of potential population heterogeneity of tickborne encephalitis and West Nile viruses using metagenomic analysis methods has revealed multiple SNVs in the viral populations of all three orthoflavivirus isolates studied, which were isolated from tissues of a single garden reed warbler. In the TBEV and WNV isolates under investigation, SNV detection rates were observed to be from 1.75 to 23.73 %.

The identified SNPs were associated with synonymous and non-synonymous single-nucleotide substitutions, both predominantly localized in the genes of viral non-structural proteins. Tomsk-PT122 TBEV (Siberian genotype) exhibited the highest SNP count (13), with a notable prevalence (23.73 % for SNP3159NS1) within the viral population. In the Tomsk-PT12 strain of TBEV of the Far Eastern genotype, SNP7150NS4b was found to be the most prevalent, with the amino acid substitution V2383L occurring at a frequency of 7.41 %. This has not been previously described for other known strains of TBEV, as well as SNP10271NS5. The smallest number of SNVs (7) was found in WNV. However, SNP1748E, SNP4434NS3b, and SVP7566NS4b had a high frequency of occurrence, from 10.55 to 19.88 % in the studied viral population.

Twenty-nine SNPs were identified in the TBEV and WNV strains under study, leading to nine viral variants with amino acid substitutions. Taken together, the results obtained highlight the significance of SNPs in ensuring the genetic diversity of orthoflaviviruses.

## Conflict of interest

The authors declare no conflict of interest.
